# Supplementary material for: Warmer environments harbor greater thermal trait diversity in moth assemblages
Source: Nat Commun. 2025 Dec 11;17:738. doi: 10.1038/s41467-025-67419-8 (PMC12820197; doi:10.1038/s41467-025-67419-8)
Supplement: Supplementary file 1 — Supplementary Information [file 41467_2025_67419_MOESM1_ESM.pdf]

Supplementary Information for
Warmer environments harbor greater thermal trait diversity in moth assemblages

Ming Liu[1,2†], Tzu-Man Hung[1,3,4†], Shipher Wu[1,5†], Mark Liu[1], Guan-Shuo Mai[1], Yi-Shin Jang[1,6], Chien-Chen Huang[4,7], Chun-Yung Hsu[1,8,9], Chia-Hsuan Wei[4], Mao-Ning Tuanmu[1], Shih-Fan Chan[1], I-Ching Chen[4]*, Sheng-Feng Shen[1,3,8]*

[1] Biodiversity Research Center, Academia Sinica, Taipei, 11529 Taiwan
[2] Department of Biology, University of Oxford, Oxford, OX1 3SZ, United Kingdom
[3] Institute of Ecology and Evolutionary Biology, National Taiwan University, Taipei, 10617 Taiwan
[4] Department of Life Sciences, National Cheng Kung University, Tainan, 70101 Taiwan
[5] National Taiwan Museum, Taipei, 10007 Taiwan
[6] Department of Atmospheric Sciences, National Taiwan University, Taipei, 10617 Taiwan
[7] School of Natural Sciences, Macquarie University, Sydney, NSW 2109 Australia
[8] International Degree Program in Climate Change and Sustainable Development, National Taiwan University, Taipei, 10617 Taiwan
[9] Social Science Research Promotion Project for Taiwan Net-Zero Pathway, National Science and Technology Council, 106214 Taiwan
[†] These authors contributed equally to this work
***** Corresponding authors: shensf@sinica.edu.tw; chenic@ncku.edu.tw

The PDF file includes:

Supplementary Methods
Supplementary Note
Supplementary Figures 1-16
Supplementary Tables 1-10
Supplementary References

Supplementary Methods

The eco-evolutionary model

Overview

Our model simulates eco-evolutionary dynamics by tracking how thermal fluctuations and species interactions determine which thermal strategies persist within a structured population. Species were generated with randomly sampled thermal performance traits and distributed across multiple patches, each initialized with potentially different species compositions. While all patches experience the same temperature at each time point, this structured population setup mimics the mosaic of local habitats in nature and allows species to persist locally even when conditions become temporarily unfavorable elsewhere. This implicit spatial structure helps buffer against the effects of short-term demographic stochasticity, enabling more realistic patterns of species survival over time.

To maintain consistency with terminology used in the main text, we define each biological unit in the model across spatial and organizational scales. A patch is a spatial unit where individual organisms reside; an individual is the basic entity subject to biological processes such as dispersal, reproduction, and mortality. A species is composed of individuals that share the same thermal performance curve, defined by three trait parameters. An assemblage refers to the full set of species with at least one surviving individual across all patches at a given time point. In addition, our model is a heuristic framework that captures environmental fluctuations across multiple time scales, but it is not intended for direct translation of a single time step into a real-world unit such as a day. This framework allows us to link individual-level processes to species-level trait dynamics and assemblage-level diversity patterns in a transparent and biologically interpretable way.

1. Generating environmental conditions

We created the thermal conditions through a two-step sampling process, where the first sampling event determines a temporary average temperature, and the second sampling event determines current temperature. In particular, the temporary average is based on mean ambient temperature and long-term thermal variability, $T_{temp} \sim Norm(\mu = T_{mean}, \sigma = T_{sd,long})$, while current temperature is based on the sampled temporary average and short-term thermal variability, $T_{current} \sim Norm(\mu = T_{temp}, \sigma = T_{sd,short})$. Importantly, the current temperature and temporary average are updated at different time scales: current temperature is updated at each time step, but the temporary average is updated less frequently—every T_{span} time steps. By this design, we can monitor the overall thermal variability, which is $T_{sd,long}^2 + T_{sd,short}^2$, and explicitly model thermal variation of different time scales. Lastly, the mean temperature, long-term variation and short-term variation are directly linked to T_{mean}, $T_{sd,long}$, and $T_{sd,short}$.

2. Calculating fecundity, which is a function of temperature

Fecundity is the number of offspring per patch for the current time step, and fecundity depends on current temperature ($T_{current}$). Based on previous literature[1,2], we assume fecundity (f_{env}) follows the relation:

$$\begin{cases} T_{current} > T_{env,max}: f_{env} = 0 \\ T_{env,max} \geq T_{current} > T_{env,opt}: f_{env} = c_{fec}\left(1 - \left(\frac{T_{current} - T_{env,opt}}{T_{env,max} - T_{env,opt}}\right)^2\right) \\ T_{env,opt} \geq T_{current}: f_{env} = c_{fec}\, exp\left(-((T_{env,opt} - T_{current})/2\sigma_{env})^2\right) \end{cases}, \tag{S1}$$

where $T_{current}$ is the current temperature, $T_{env,max}$ is the maximal viable temperature, $T_{env,opt}$ is the most-fecund temperature, c_{fec} is the scaling coefficient, σ_{env} is the coefficient controlling the decay speed when temperature is lower than optimal, and exp represents an exponential function with the base of Euler's number.

Our fecundity design can be viewed as an outcome of the biochemical activities of most enzymes. Based on the effects of protein denaturation, we assume no organism can produce any offspring when the temperature is

beyond $T_{env,max}$, and there is a sharp decrease in fecundity once temperature passes $T_{env,opt}$. On the contrary, when temperature is below $T_{env,opt}$, the modeled organism may still produce some offspring, and thus there is a gradual decline in fecundity.

3. Calculating the thermal performance for each species

As our goal is modeling environmental (abiotic) filtering effects in functional trait space for assemblages, we stochastically generate each species' thermal performance parameters. There are three parameters for each species: (i) the optimal temperature, (ii) the distance between maximal viable temperature and the optimum, (iii) the steepness of decaying performance when temperature is below optimum. We assume each parameter follows a normal distribution:

$$\begin{cases} T_{spc,opt} \sim Norm(\mu_{opt}, \sigma_{opt}) \\ \left(T_{spc,max} - T_{spc,opt}\right) \sim Norm(\mu_{inc}, \sigma_{inc}), \\ \sigma_{spc} \sim Norm(\mu_{sgm}, \sigma_{sgm}) \end{cases} \tag{S2}$$

where μ and σ are the average and standard deviation of each distribution. The values are randomly picked and do not qualitatively change the results. To maintain biological meaning for all parameters, we resample the parameters when (1) $\left(T_{spc,max} - T_{spc,opt}\right)$ or (2) σ_{spc} is negative.

In addition to randomly generating the thermal performance parameters, we standardize the area under each species' performance curve to ensure a standardized competition among all species (this assumption is relaxed in Supplementary Fig. 1). In particular, we calculate the scaling coefficient based on the sum of area,

$$c_{pf} = \frac{\frac{2}{3}\times\mu_{inc}+\mu_{sgm}\sqrt{\pi}}{\frac{2}{3}\times\left(T_{spc,max}-T_{spc,opt}\right)+\sigma_{spc}\sqrt{\pi}}. \tag{S3}$$

Each component of the coefficient is obtained from integration of each region of performance, where $\frac{2}{3}\times\left(T_{spc,max} - T_{spc,opt}\right)$ is from $\int_{T_{spc,opt}}^{T_{spc,max}} 1 - (\frac{T_{current}-T_{spc,opt}}{T_{spc,max}-T_{spc,opt}})^2\, dT$, and $\sigma_{spc}\sqrt{\pi}$ is from $\int_{-\infty}^{T_{spc,opt}} exp\,(-((T_{spc,opt} - T_{current})/2\sigma_{spc})^2)dT$. The numerator simply replaces the results of integration with the average of each normal distribution in expression S2.

Combining the elements of the model described above, we find the thermal performance of each species at current temperature ($T_{current}$) as:

$$\begin{cases} T_{current} > T_{spc,max}: f_{spc,T} = 0 \\ T_{spc,max} \geq T_{current} > T_{spc,opt}: f_{spc,T} = c_{pf}(1 - \left(\frac{T_{current}-T_{spc,opt}}{T_{spc,max}-T_{spc,opt}}\right)^2) \\ T_{env,opt} \geq T_{current}: f_{spc,T} = c_{pf}(exp\left(-\left(\frac{T_{spc,opt}-T_{current}}{2\sigma_{spc}}\right)^2\right)) \end{cases}. \tag{S4}$$

Although both fecundity and thermal performance share the same design (expressions S1 and S4), their properties differ in two ways: (1) the fecundity design has a constant and defined relationship to temperature while thermal performance is stochastically sampled from expression S2; (2) fecundity affects how many new individuals are produced in each time step, while thermal performance determines how the new individuals are allocated between species within the same patch.

4. Reproduction

We assume the allocation of offspring consists of two parts: density-dependent and density-independent. The

probability of density-dependent reproduction is controlled by a type-II functional response, $p_{compete} = \frac{w}{1+w}$, where w is the summed species weights in the focal patch,

$$w_i = \sum_{j=1}^{n_{spcs}} \text{mininum} \left(c_{weight} \times n_{i,j}, 1\right). \tag{S5}$$

In expression S5, n_{spcs} is the number of species, $mininum$ is the function that output the minimum of values within, c_{weight} is the coefficient for weighting and $n_{i,j}$ is the density of species j at patch i. The implementation of minimal function here is to discount rare species that has very few individuals (i.e., when $c_{weight} \times n_{i,j}$ is less than 1). Taken together, we assume that the probability of density-dependent reproduction ($p_{compete}$) is a function that saturates as the sum of density increases, a relationship that is widely documented in nature[3,4].

When density-dependent reproduction takes place, the new individual is allocated to a species according to the relative competitiveness of all species in the focal patch. The relative competitiveness is defined as $f_{spc,i} = f_{spc,T} \times n_{i,j}$, where i denotes focal patch and j denotes focal species. In contrast, when density-independent reproduction takes place, we randomly pick a species that still exists in the focal patch and allocate the new individual to it according to the probability of its thermal performance ($f_{spc,T}$). If it fails, we repeat the process a total for N_{samp} times; no species reproduces when all attempts fail. In other words, density-independent reproduction still depends on thermal performance but does not depend on the density of species. Importantly, we sum the new individuals for each species over all patches and disperse them after the mortality process is complete.

5. Mortality

For simplicity, we assume that mortality does not change with thermal conditions. Therefore, mortality is a fixed probability, p_{mort}, for all species at individual level across the whole simulation.

6. Allocating the offspring

New-born individuals of each species can freely disperse to any patch, and we randomly pick a patch and add one adult to the chosen patch for each offspring.

7. Creating new species

In our main model, we simulate the introduction of new species via immigration, analogous to allopatric speciation. This approach was chosen because it facilitates efficient exploration of the thermal trait space and accelerates the system's convergence to evolutionary steady states, particularly under fluctuating environmental conditions. At each time step, new species are introduced with a probability defined by the immigration rate (p_{immi}) , provided that the assemblage has not yet reached the maximum number of species allowed (N_{spcs_max}) . Each immigrant species is generated using the same thermal trait initialization process described in Section 3. Once generated, the species is introduced $N_{patch\_newspcs}$ randomly selected patches, each seeded with N_{ini} individuals. These new species then participate in the full eco-evolutionary dynamics of the system. To ensure the assemblage does not become saturated, we conservatively calibrated the immigration rate by selecting the smallest value that consistently allowed species diversity to stabilize across environmental scenarios without overwhelming the system.

To test the robustness of this assumption, we implemented an alternative mechanism for generating diversity based on sympatric speciation via mutation. In this variant, new species arise from existing species at the same rate as immigration (p_{immi}). Each new species is introduced with the same initial abundance and random spatial distribution as in the immigration scenario. The key difference lies in how thermal traits are assigned. Instead of independent sampling as in the immigration design, the new species inherits its trait structure from a randomly chosen parent species, with variation introduced by Gaussian noise:

$$\begin{cases} T_{mut\_spc,opt} \sim Norm(\mu_{old\_spc,opt}, \sigma_{mut}) \\ (T_{mut\_spc,max} - T_{mut\_spc,opt}) \sim Norm(\mu = T_{old\_spc,max} - T_{old\_spc,opt}, \sigma_{mut}), \\ \sigma_{mut\_spc} \sim Norm(\mu = \sigma_{old\_spc}, \sigma_{mut}) \end{cases} \quad \text{(S6)}$$

where subscript old_spc denotes the sampled existing species, and mut_spc denotes the newly created species through mutation. In addition, we set the standard deviation for all three traits to $\sigma_{mut} = 1.0$ to avoid making some traits more variable than others. This alternative produced consistent results across the three environmental hypotheses (see Supplementary Fig. 9), indicating that our conclusions are not sensitive to the mode of diversity generation.

8. *Code execution*

We initialize the assemblage by setting the number of patches to a large number (N_{patch}), to reduce demographic stochasticity. Each patch starts with the same number of individuals per species (N_{ini}), and the number of species per patch is also uniform. As we are interested in examining the favorability and variability hypotheses, we hold the variation constant and change mean temperature in the former case; hold the average constant and change variation in the latter case. For each environmental setting, we run N_{rep} repeating trials to increase the pool of selected species. In each simulation trial, we ran for 100,000 time steps with 5 randomly generated species in the initial assemblage.

9. *Data collection*

Because of the nature of exponential function, the lower end of thermal performance always goes to negative infinity, and it is not meaningful to define the critical temperature as when thermal performance reaches zero. Therefore, we define the CTmin and CTmax for each species as the temperatures at which their thermal performance is 5% of the optimal thermal performance. Specifically, they can be numerically solved from expression S4,

$$\begin{cases} 1 - \left(\frac{CTmax - T_{spc,opt}}{T_{spc,max} - T_{spc,opt}}\right)^2 = 0.05 \\ exp\left(-\left(\frac{T_{spc,opt} - CTmin}{2\sigma_{spc}}\right)^2\right) = 0.05 \end{cases}, \quad \text{(S7)}$$

where $CTmax \approx 0.025 \times T_{spc,opt} + 0.975 \times T_{spc,max}$, and $CTmin \approx T_{spc,opt} - 3.46 \times \sigma_{spc}$. We record the CTmin and CTmax of each surviving species at the end of each simulation.

Supplementary Note

Disentangling competitive effects from environmental conditions

We compared the mean species density under identical environmental settings to isolate the effects of interspecific competition (Supplementary Figs. 10-12). Specifically, species were grouped based on their critical thermal minimum (CTmin) and maximum (CTmax), with the mean species density of each group visualized in a heat map. When competition was present, we observed a distinct region where mean species density peaked (e.g., CTmax = 28 and CTmin = 12 in Supplementary Fig. 10c). This pattern suggests that a narrow range of CTmin-CTmax combinations yields the most competitive advantage in the assemblage.

In contrast, in the absence of competition, two distinct patterns of species density emerged: a gradual decline with increasing CTmin and an absence-presence shift with rising CTmax (e.g., Supplementary Fig. 10, d-f). These results can be explained by the shape of the thermal performance curve. On the lower end, performance declines exponentially, whereas, on the higher end, it shows a quadratic decline. Consequently, species with a low CTmax have a performance that quickly drops to zero, while species with high CTmin maintain a small but consistent performance.

Both patterns of mean species density, with and without competition, holds when varying mean ambient temperature (Supplementary Fig. 10), short-term variability (Supplementary Fig. 11), and long-term variability (Supplementary Fig. 12). In other words, the three environmental properties only change the pattern quantitatively. In particular, increasing mean temperature boosts the overall species density as a whole; increasing short-term variability makes the transition from high to low density smoother; increasing long-term variability enhances the stochasticity. These finer differences reflect our model's environmental fluctuation design. Although our model does not incorporate within-generation phenotypic plasticity, the distinction between plastic responses and long-term trait evolution remains conceptually important. Classical plasticity models[1,2] focus on how environmental predictability favors plasticity, whereas our framework explores the selection of fixed thermal traits. Future integration of these perspectives may offer broader insights into physiological adaptation under environmental variability.

Supplementary Figures

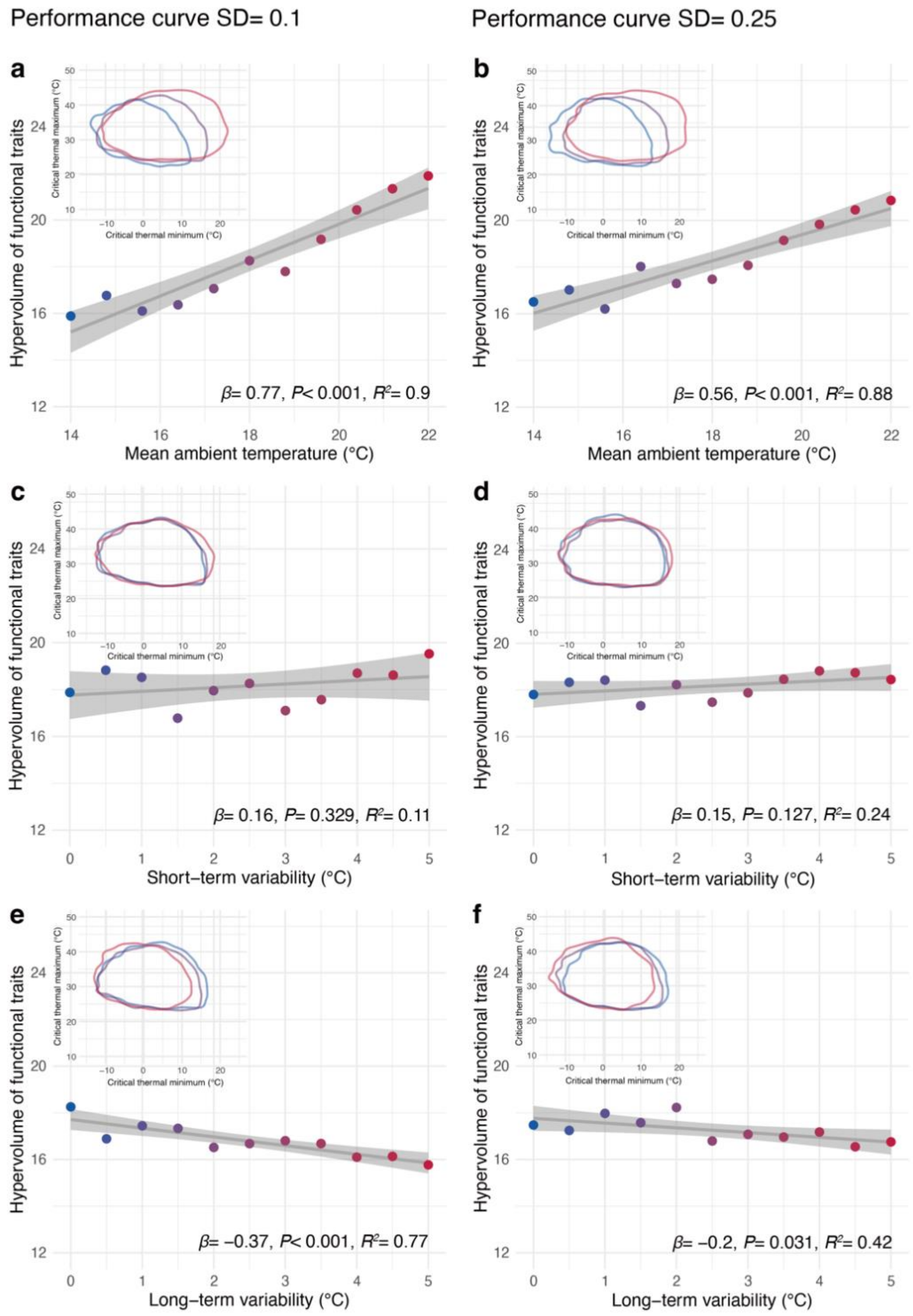

Supplementary Fig. 1. Model results with weaker correlations. As in Figure 2, panels show the effects of mean ambient temperature (**a**, **b**), short-term variability (**c**, **d**) and long-term variability (**e**, **f**) on the hypervolume of assemblage thermal traits. The main model assumes all species have identical areas of thermal performance curves (i.e., a strong correlation between the width and height). Here we relaxed this assumption here by rescaling the area with a random number from a normal distribution, with mean of 1 and standard deviation of 0.1 (**a**, **c**, **e**) or 0.25 (**b**, **d**, **f**). Lines are linear fits with 95% confidence bands, *P*-values are from two-sided *F*-tests.

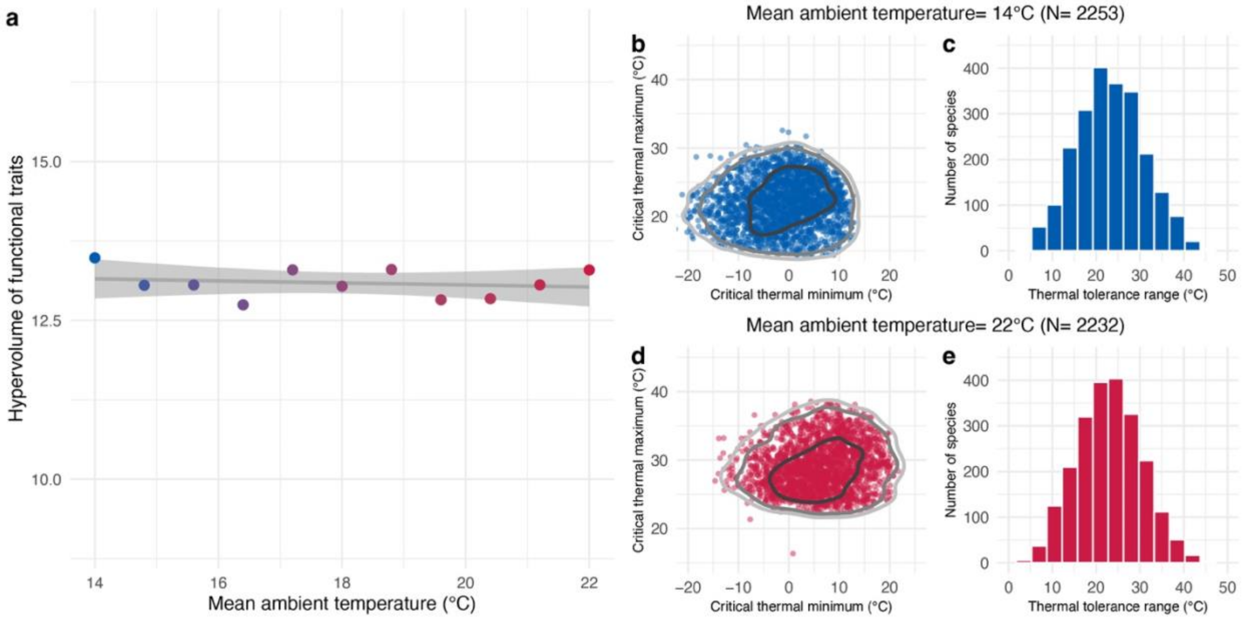

Supplementary Fig. 2. Model results with no productivity assumption. a Hypervolume of thermal traits against mean ambient temperature (β= -0.016, P= 0.591, R^2= 0.03, two-sided F-test). **b** Distribution of critical thermal limits when the mean ambient temperature is 14 °C (correspond to Fig. 3**a**). **c** Histogram of species numbers along TTrange at 14°C. **d** Distribution of critical thermal limits when the mean ambient temperature is 22 °C (correspond to Fig. 3**a**). **e** Histogram of species numbers along TTrange at 22°C. Shaded area in **a** represent the 95% confidence interval for the fitted regression line.

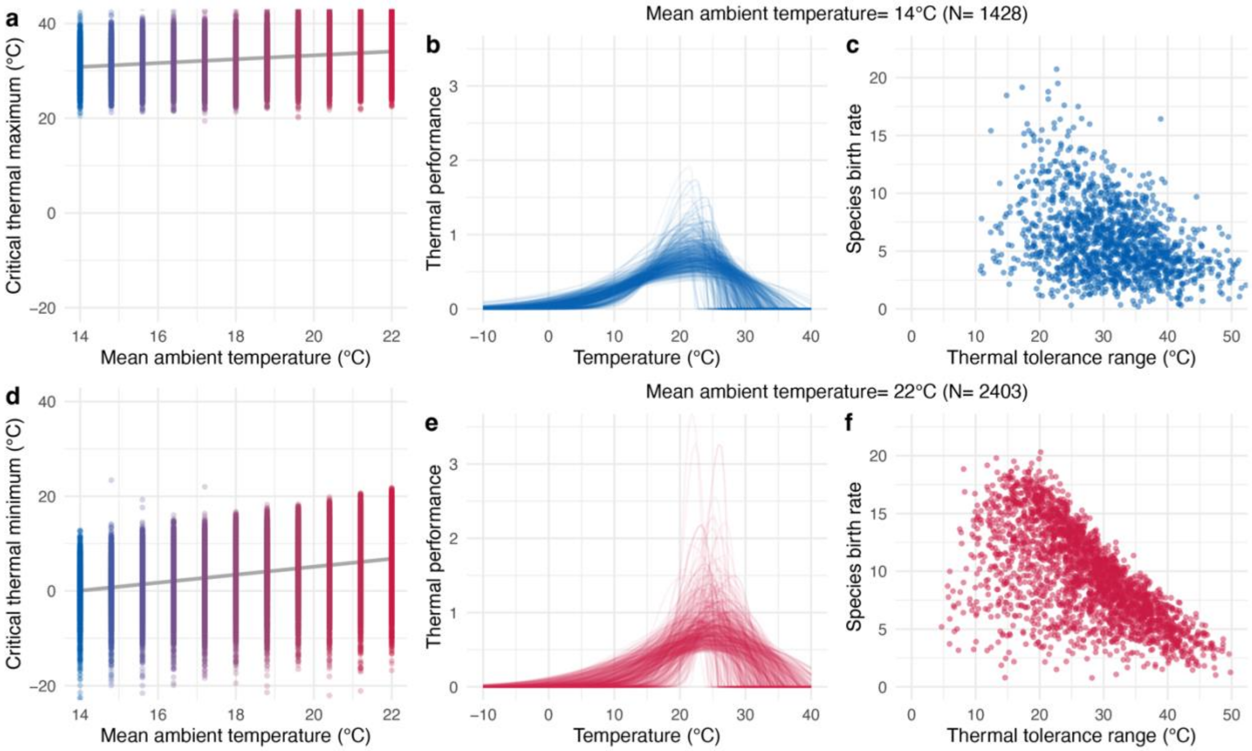

Supplementary Fig. 3. Additional analyses for favorability hypothesis in the eco-evolutionary model. a Critical thermal maximum of each species ($\boldsymbol{\beta}$= 0.407, $\boldsymbol{P}$< 0.001, $\boldsymbol{R^2}$= 0.06**, two-sided F-test**). **b** Shape of thermal performance curves in the assemblage under the lowest ambient temperature setting (500 random samples from the assemblage). **c** Birth rate of each species in the same assemblage. Birth rate is estimated from the number of new-born individuals over the last 100 timesteps of the 10^5-step simulation. **d** Critical thermal minimum of each species ($\boldsymbol{\beta}$= 0.841, $\boldsymbol{P}$< 0.001, $\boldsymbol{R^2}$= 0.09, two-sided F-test). **e-f** Same as **b** and **c**, but for the assemblage under the highest ambient temperature setting. The assemblages presented here are identical to those in Fig. 2**a** and **b**.

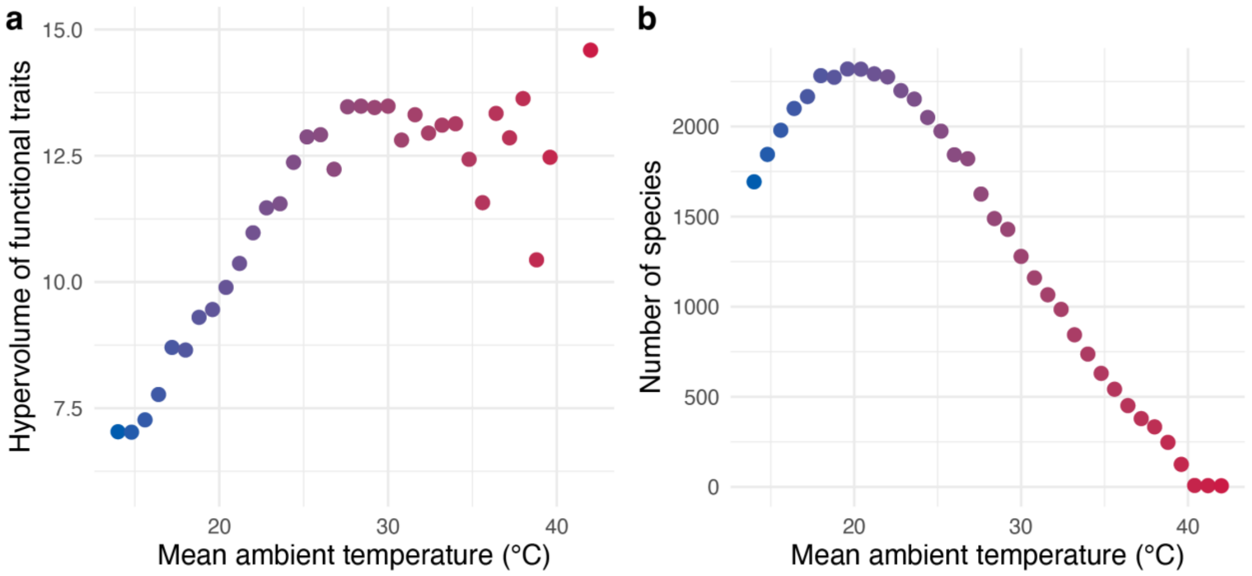

Supplementary Fig. 4. Effects of mean ambient temperature on thermal trait diversity and number of species. Variation in the hypervolume of functional traits (**a**) and the number of species (**b**) across different mean ambient temperatures.

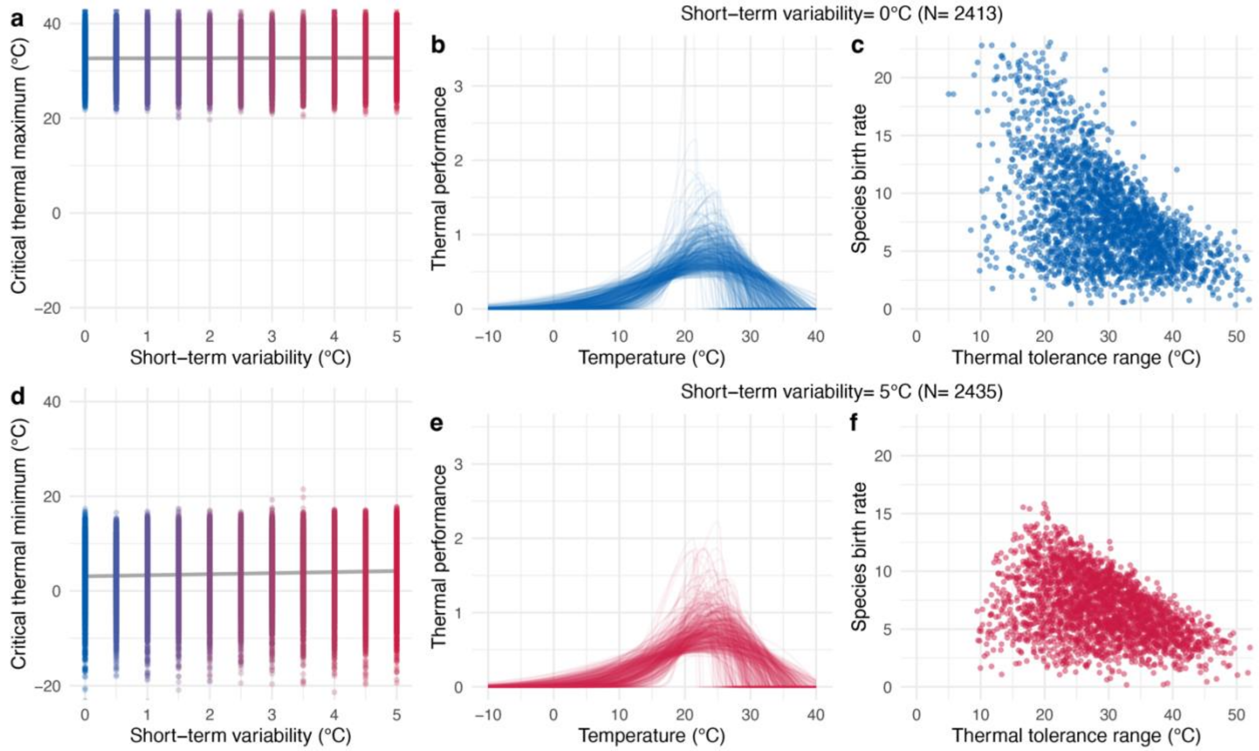

Supplementary Fig. 5. Additional analyses for the short-term variability hypothesis. **a** Critical thermal maximum of each species (β= 0.022, P= 0.151, R^2< 0.01, two-sided F-test). **b** Shape of thermal performance curves in the assemblage under the lowest thermal variability setting. **c** Birth rate of each species in the same assemblage. Birth rate is estimated from the number of new-born individuals over the last 100 timesteps of the 10^5-step simulation. **d** Critical thermal minimum of each species (β= 0.223, P< 0.001, R^2< 0.01, two-sided F-test). **e-f** Same as **b** and **c** but for the assemblage under the highest short-term variability setting. The assemblages presented here are identical to those in Fig. 1**c**, **d** and Fig. 2**e-h**.

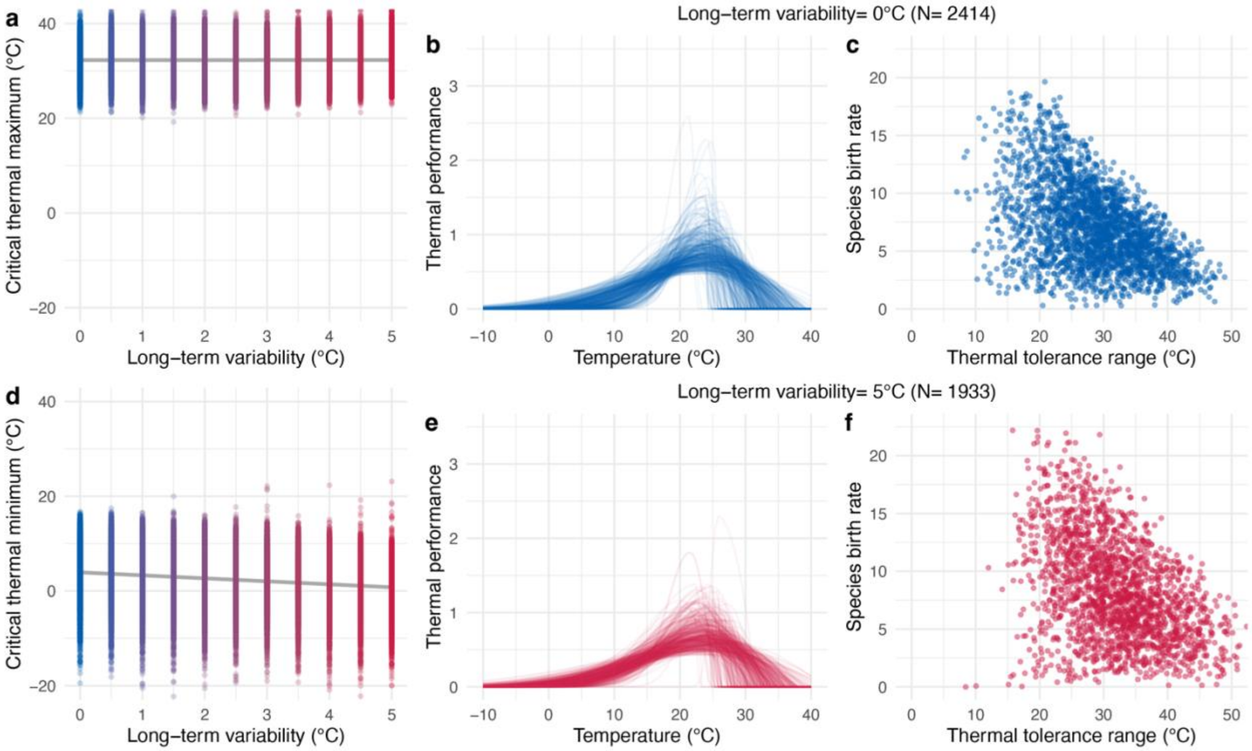

Supplementary Fig. 6. Additional analyses for the climatic variability hypothesis. a Critical thermal maximum of each species (β= 0.003, P= 0.835, R^2< 0.01, two-sided F-test). **b** Shape of thermal performance curves in the assemblage under the lowest thermal variability setting. **c** Birth rate of each species in the same assemblage. Birth rate is estimated from the number of new-born individuals over the last 100 timesteps of the 10^5-step simulation. **d** Critical thermal minimum of each species (β= -0.631, P< 0.001, R^2= 0.03, two-sided F-test). **e-f** Same as **b** and **c** but for the assemblage under the highest short-term variability setting. The assemblages presented here are identical to those in Fig. 1**e**, **f** and Fig. 2**i-l**.

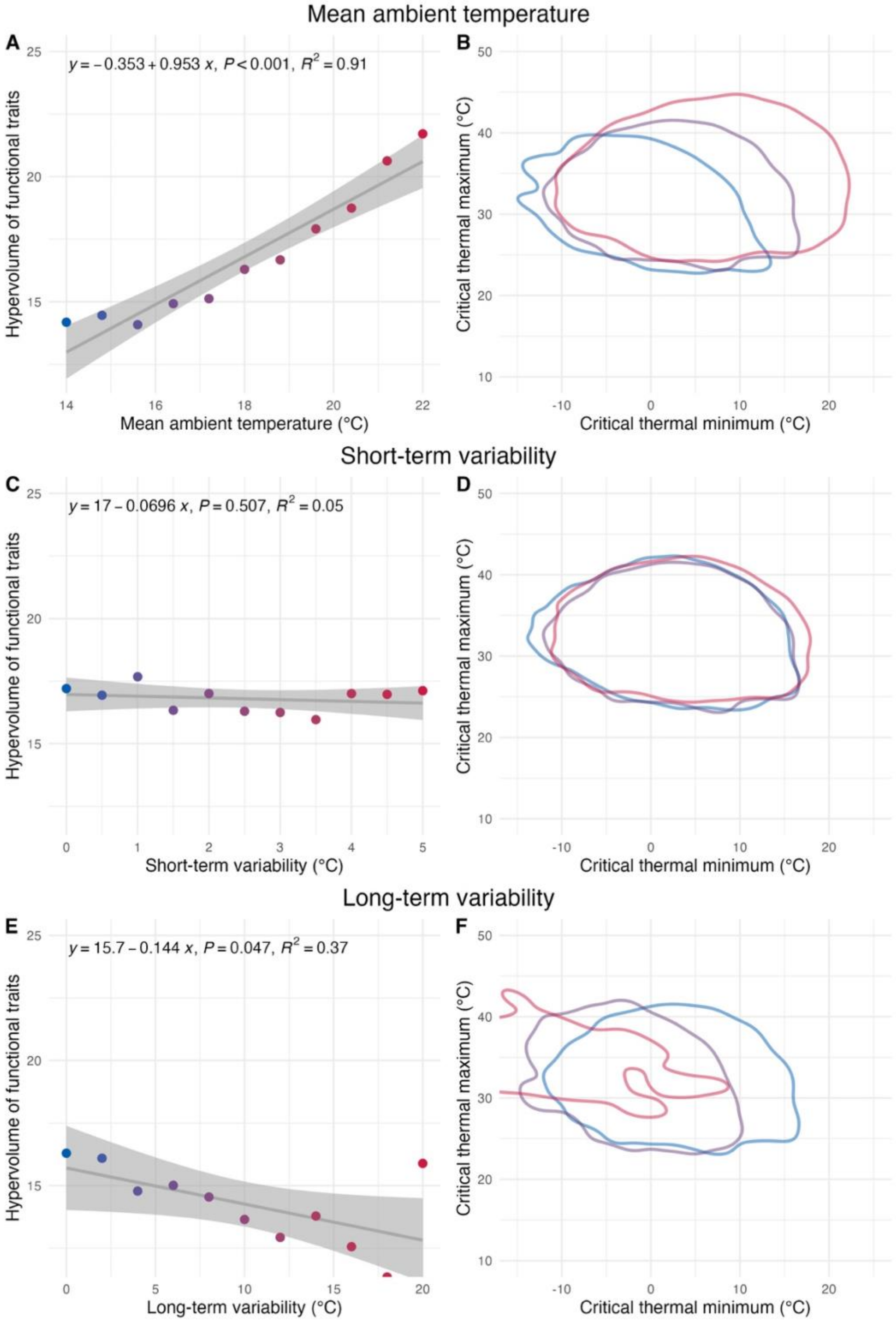

Supplementary Fig. 7. Model results are robust against alternative and deterministic long-term variation design. Here, instead of sampling from a normal distribution every T_{span}, T_{temp} is following a sine wave with period of 500 time-steps. As in Figure 2, panels show the effects of mean ambient temperature (**a**, **b**), short-term variability (**c**, **d**) and long-term variability (**e**, **f**) on the hypervolume of assemblage thermal traits. Panel **a**, **c** and **e** show the relationship between the hypervolume of functional thermal traits and each climatic property, with fitted linear regressions (solid lines) and 95% confidence intervals (grey shading), P-values are from two-sided F-tests. Panels **b**, **d** and **f** show the 95% quantile contours of critical thermal limits for three assemblages at low (1st; blue), middle (6th; purple) and high (11th; red) levels of the corresponding climatic variable.

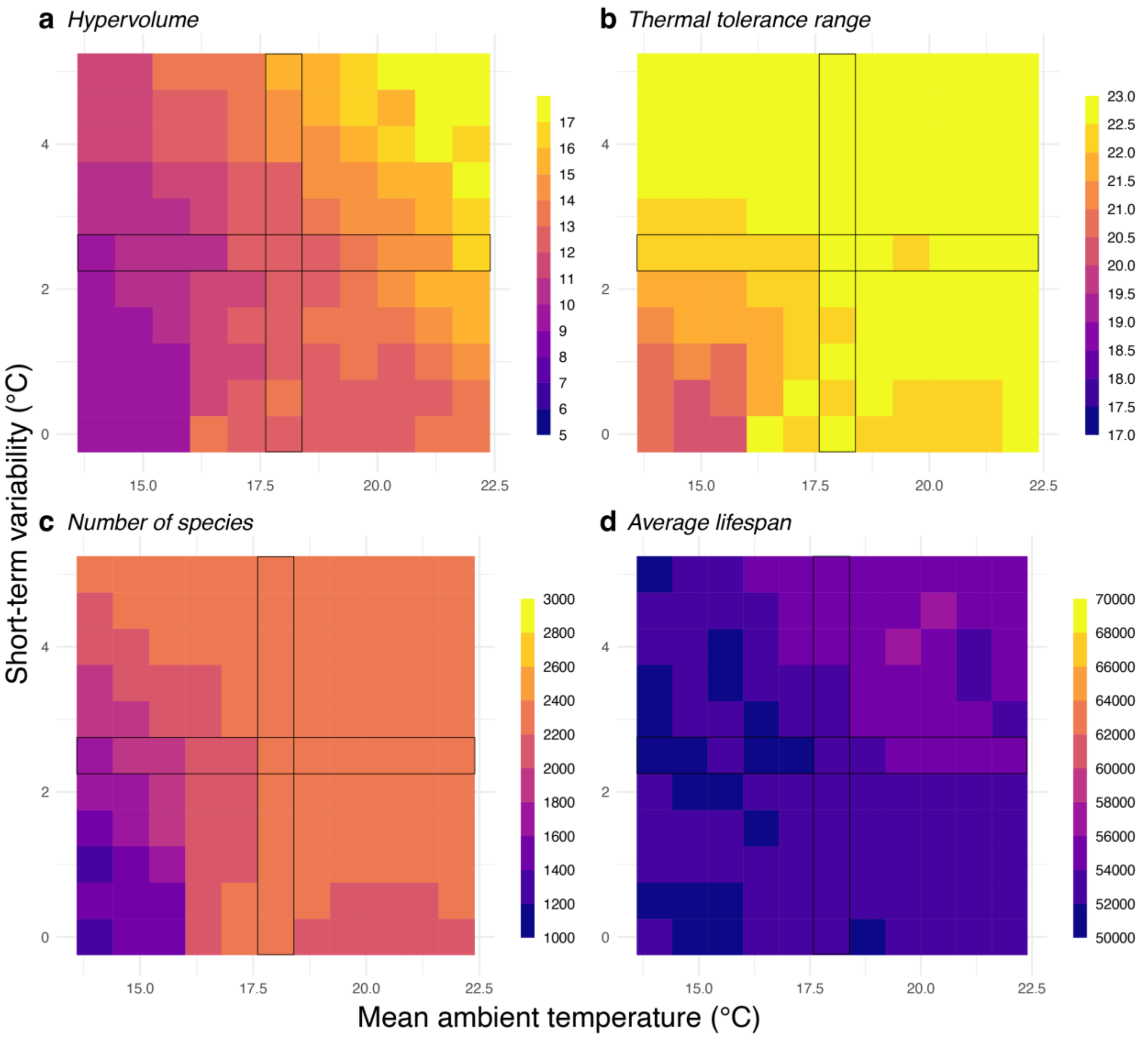

Supplementary Fig. 8. Broader exploration across mean ambient temperature and thermal variability for the eco-evolutionary model. **a** Hypervolume of assemblage thermal traits under each combination of mean ambient temperature (x-axis), and thermal variability (y-axis). **b** Average thermal tolerance range of each assemblage. **c** Number of surviving species at the end of simulations. **d** Lifespan of each surviving species. The black frames in each panel indicate the parameters used in Fig. 2, where the horizontal frame tests the favorability hypothesis and the vertical frame tests the short-term variability hypothesis. Long-term variability is set to zero in this figure.

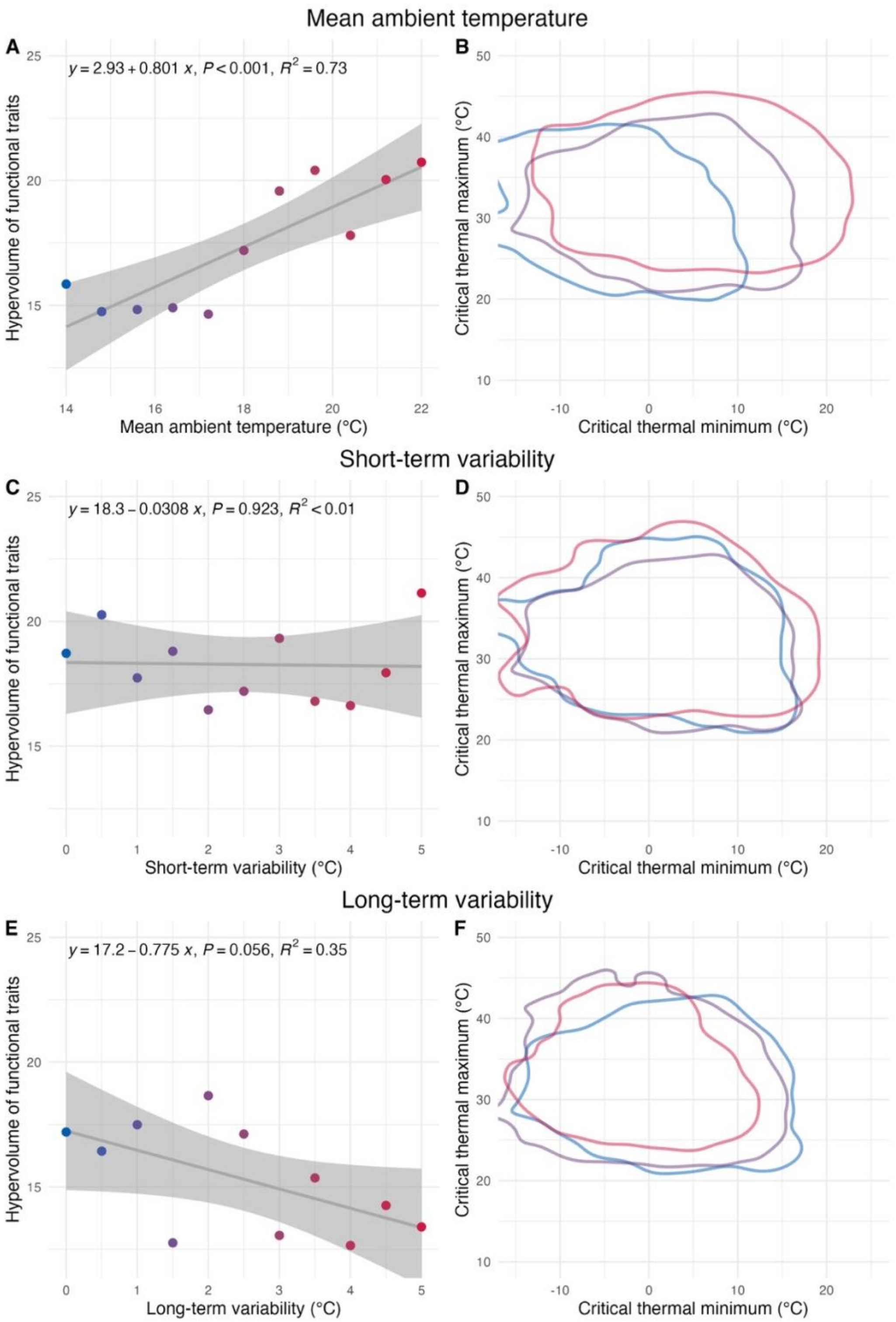

Supplementary Fig. 9. Model results are robust against alternative and sympatric speciation design. Here, new species are created through sampling currently viable species and make small trait mutations from them. See more details in Supplementary Methods section 7, *creating new species*. As in Figure 2, panels show the effects of mean ambient temperature (**a**, **b**), short-term variability (**c**, **d**) and long-term variability (**e**, **f**) on the hypervolume of assemblage thermal traits. Panel **a**, **c** and **e** show the relationship between the hypervolume of functional thermal traits and each climatic property, with fitted linear regressions (solid lines) and 95% confidence intervals (grey shading), *P*-values are from two-sided F-tests. Panels **b**, **d** and **f** show the 95% quantile contours of critical thermal limits for three assemblages at low (1st; blue), middle (6th; purple) and high (11th; red) levels of the corresponding climatic variable.

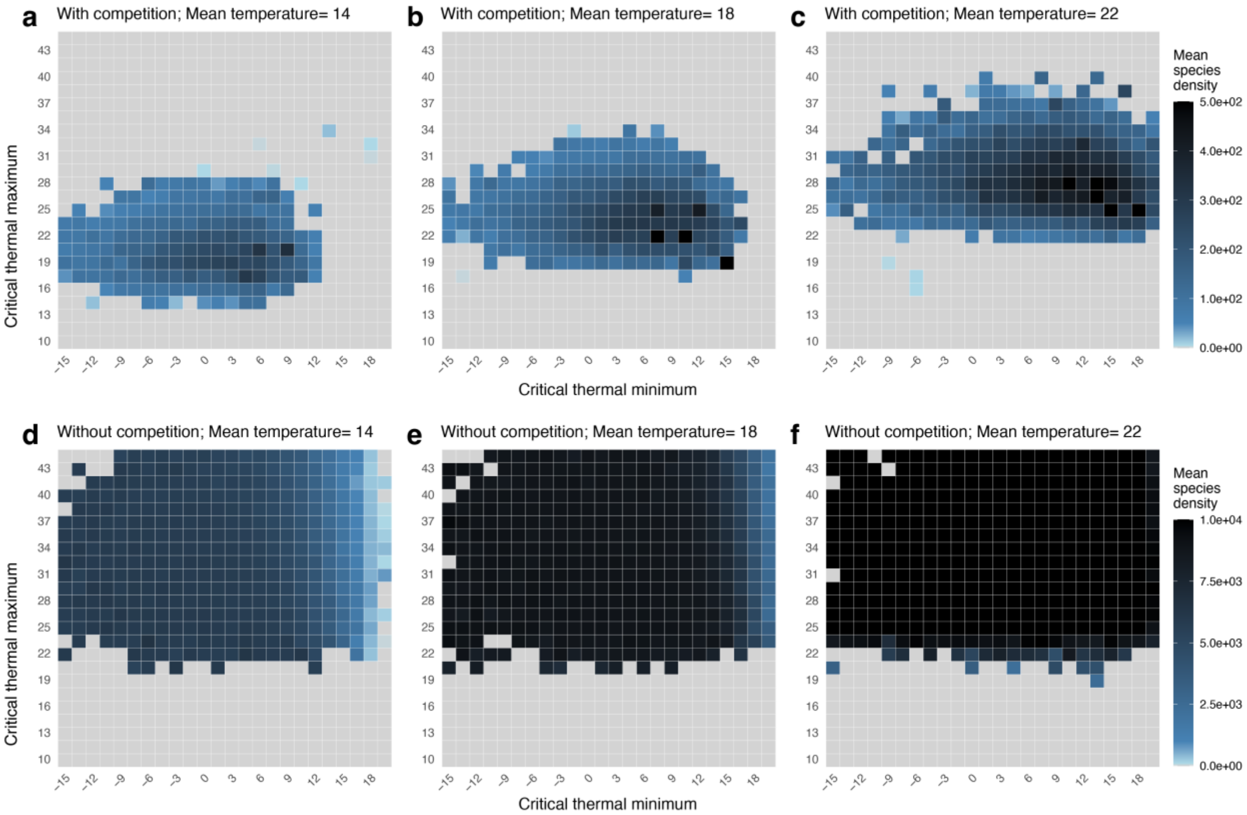

Supplementary Fig. 10. Effect of competition on mean species density along mean temperature. a-c Mean species density, the average density of each species in the CTmin-CTmax combination, under 14°C (**a**), 18°C (**b**) and 22°C (**c**) mean temperature with species competition. **d-f** Mean species density under 14°C (**d**), 18°C (**e**) and 22°C (**f**) mean temperature without species competition. Species competition is present in all figures except Supplementary Figs. 10-12, **d-f**. The removal of competition is achieved by simulating one species at a time without immigration (10,000 repeats in each panel). Short-term variability is set to 2.5 and long-term variability is set to 0.

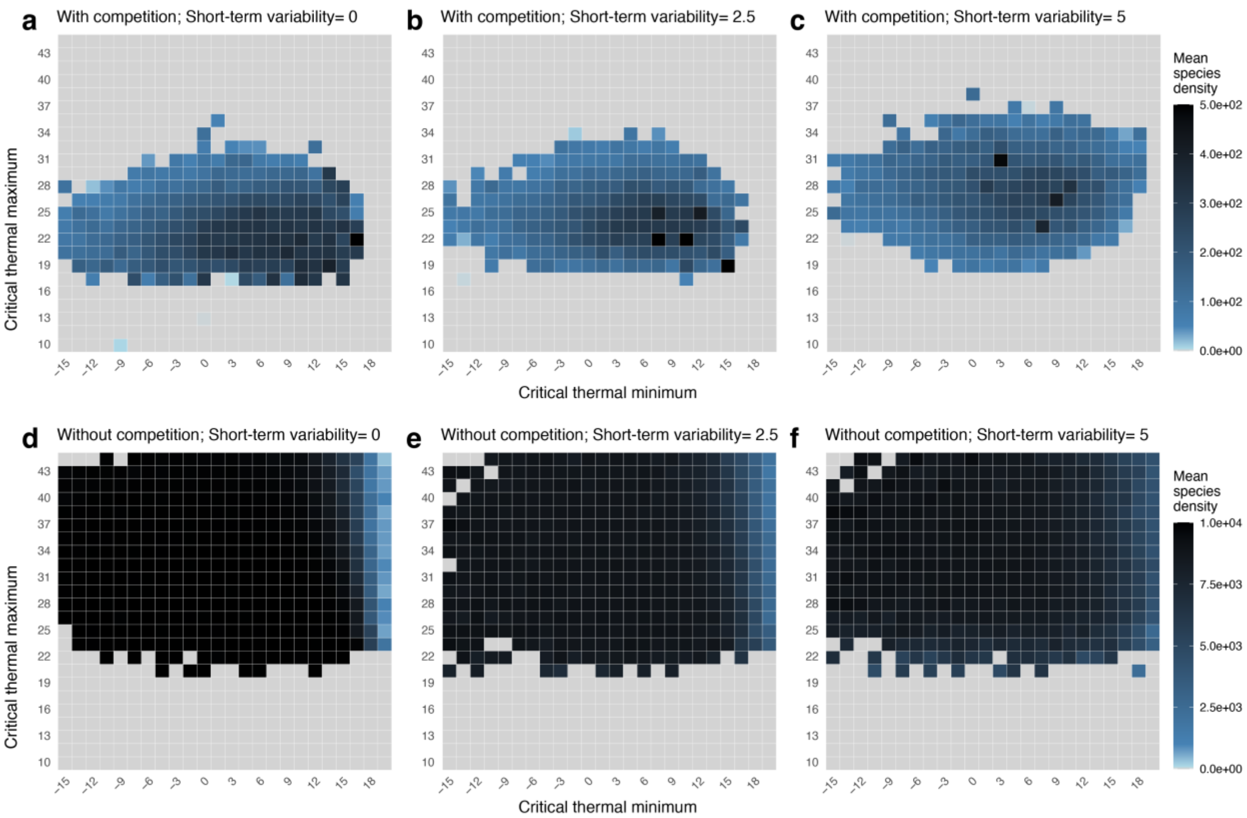

Supplementary Fig. 11. Effect of competition on mean species density along short-term variability. a-c Mean species density under 0°C (**a**), 2.5°C (**b**) and 5°C (**c**) short-term variability with species competition. **d-f** Mean species density under 0°C (**d**), 2.5°C (**e**) and 5°C (**f**) short-term variability without species competition. Species competition is present in all figures except Supplementary Figs. 10-12, **d-f**. The removal of competition is achieved by simulating one species at a time without immigration (10,000 repeats in each panel). Mean temperature is set to 18 and long-term variability is set to 0.

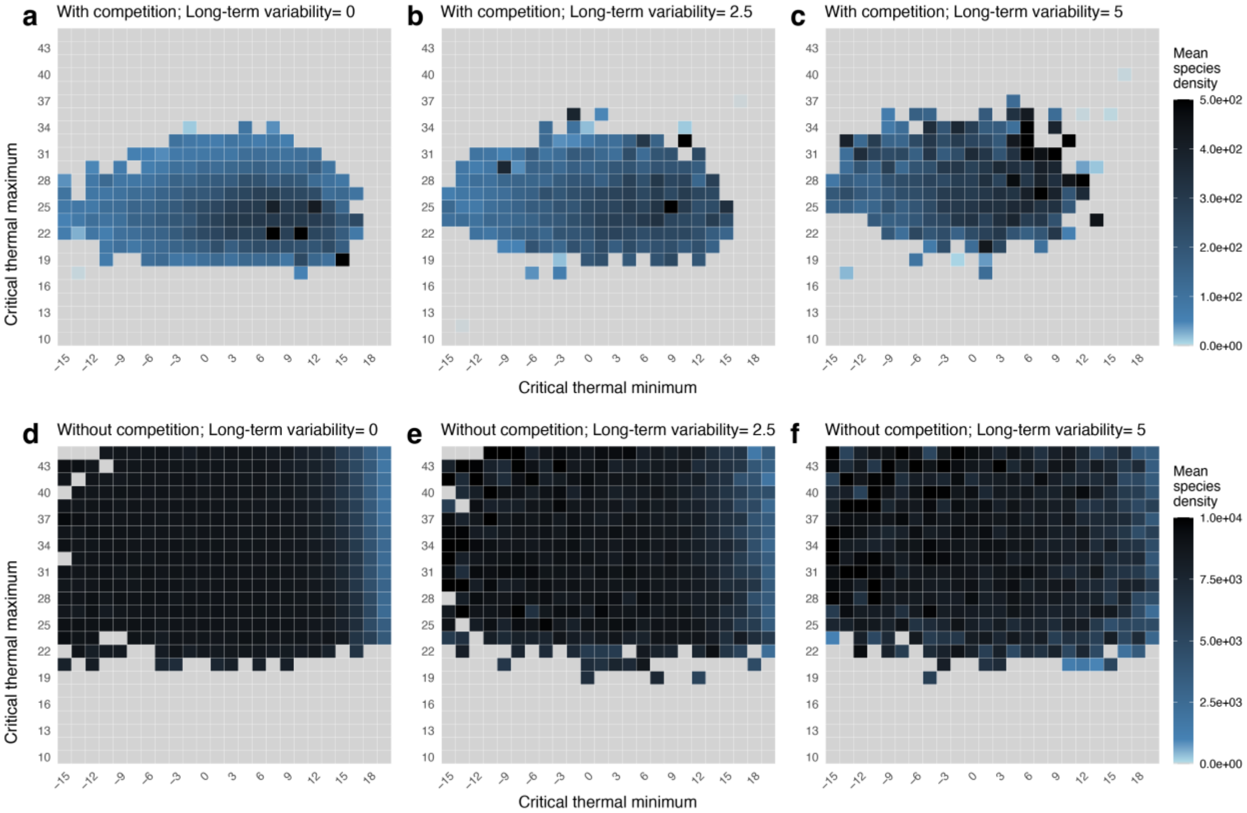

Supplementary Fig. 12. Effect of competition on mean species density along long-term variability. a-c Mean species density under 0°C (**a**), 2.5°C (**b**) and 5°C (**c**) long-term variability with species competition. **d-f** Mean species density under 0°C (**d**), 2.5°C (**e**) and 5°C (**f**) long-term variability without species competition. Species competition is present in all figures except Supplementary Figs. 10-12, **d-f**. The removal of competition is achieved by simulating one species at a time without immigration (10,000 repeats in each panel). Mean temperature is set to 18 and short-term variability is set to 2.5.

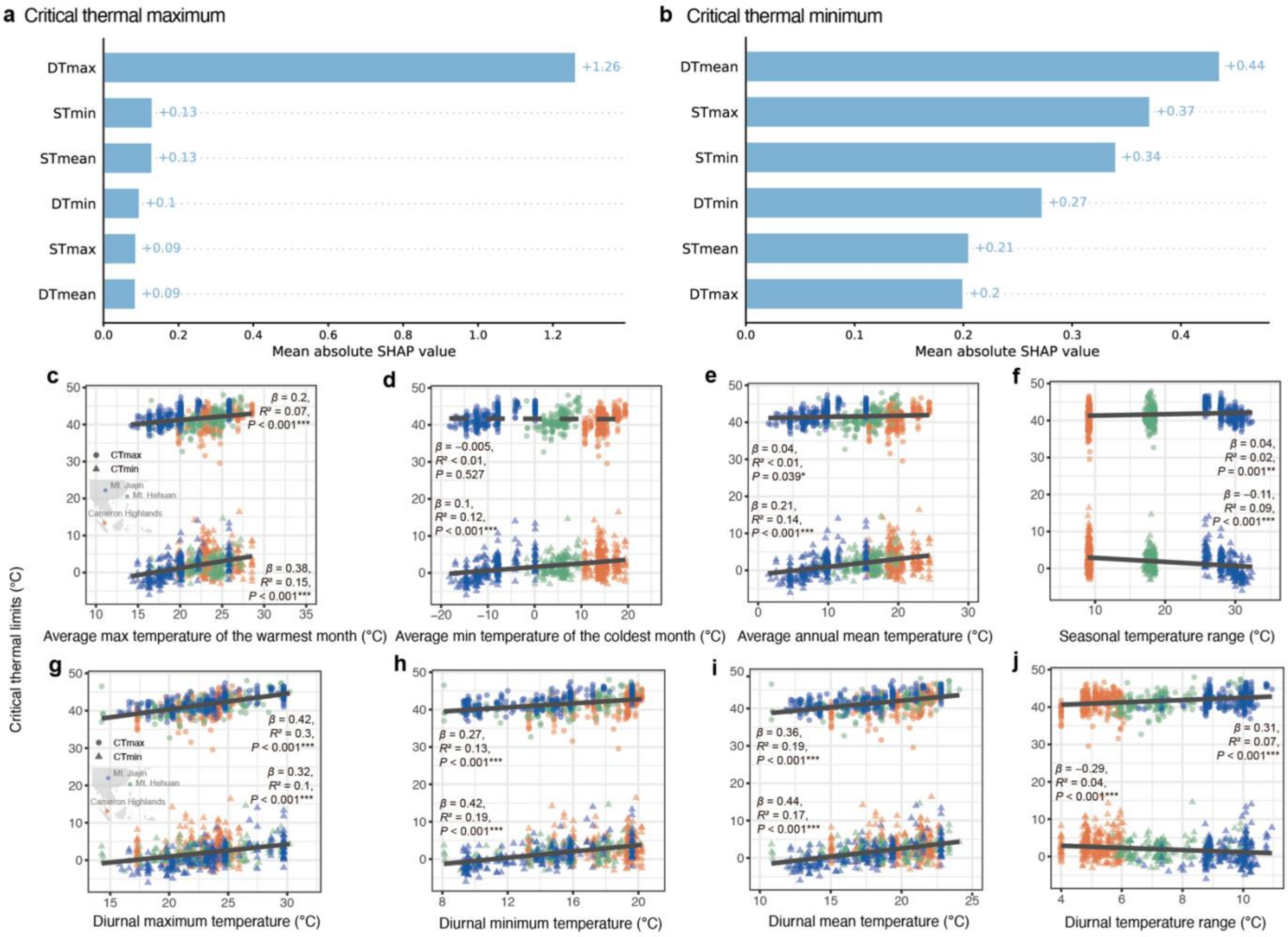

Supplementary Fig. 13. Relationships between critical thermal limits and ambient seasonal temperature. **a-b** Shapley additive explanation (SHAP) value of features of the random forest models (repeated 10 times, each with 500 randomly generated trees) of critical thermal maximum (CTmax, **a**), critical thermal minimum (CTmin, **b**), ranked by their overall impact as denoted by their mean absolute SHAP value, which reflects overall influence on predicted thermal limits of each feature. **c-j** Critical thermal maximum and minimum in relation to average max temperature of the warmest month (STmax, **c**), average min temperature of the coldest month (STmin, **d**), average annual mean temperature (STmean, **e**), seasonal temperature range (STR, **f**), diurnal maximum temperature (DTmax, **g**), diurnal minimum temperature (DTmin, **h**), diurnal mean temperature (DTmean, **i**), diurnal temperature range (DTR, **j**). In **c-j**, solid lines indicate significant relationships (P < 0.05, two-sided Type II Wald chi-square tests), dashed lines indicate insignificant relationships (P > 0.05, two-sided Type II Wald chi-square tests), and shaded areas represent the 95% confidence interval of the regression line. Source data files are provided in a permanent Zenodo repository under the accession code 17409650. (https://doi.org/10.5281/zenodo.17409650)

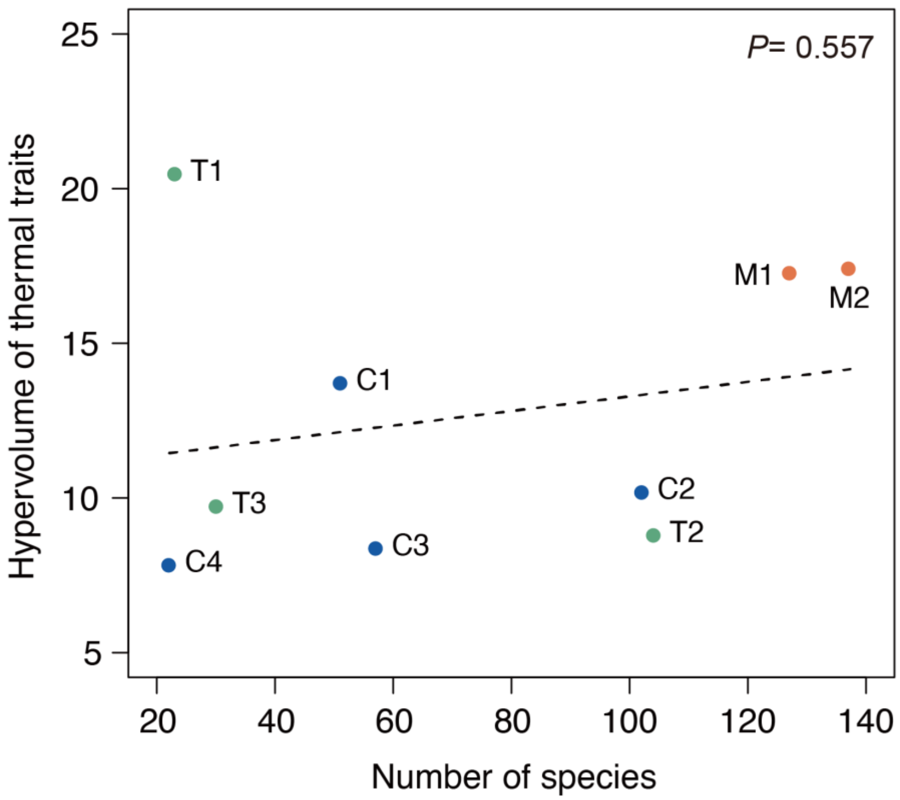

Supplementary Fig. 14. Hypervolume of thermal traits in relation to number of observed species. Dashed lines indicate insignificant relationships (P > 0.05, two-sided Type II Wald chi-square tests). Texts next to points indicates the assemblage numbers in Fig. 4**a**. Each hypervolume was averaged over 100 iterations, with each iteration randomly bootstrapping 20 species from each assemblage. Source data files are provided in a permanent Zenodo repository under the accession code 17409650. (https://doi.org/10.5281/zenodo.17409650)

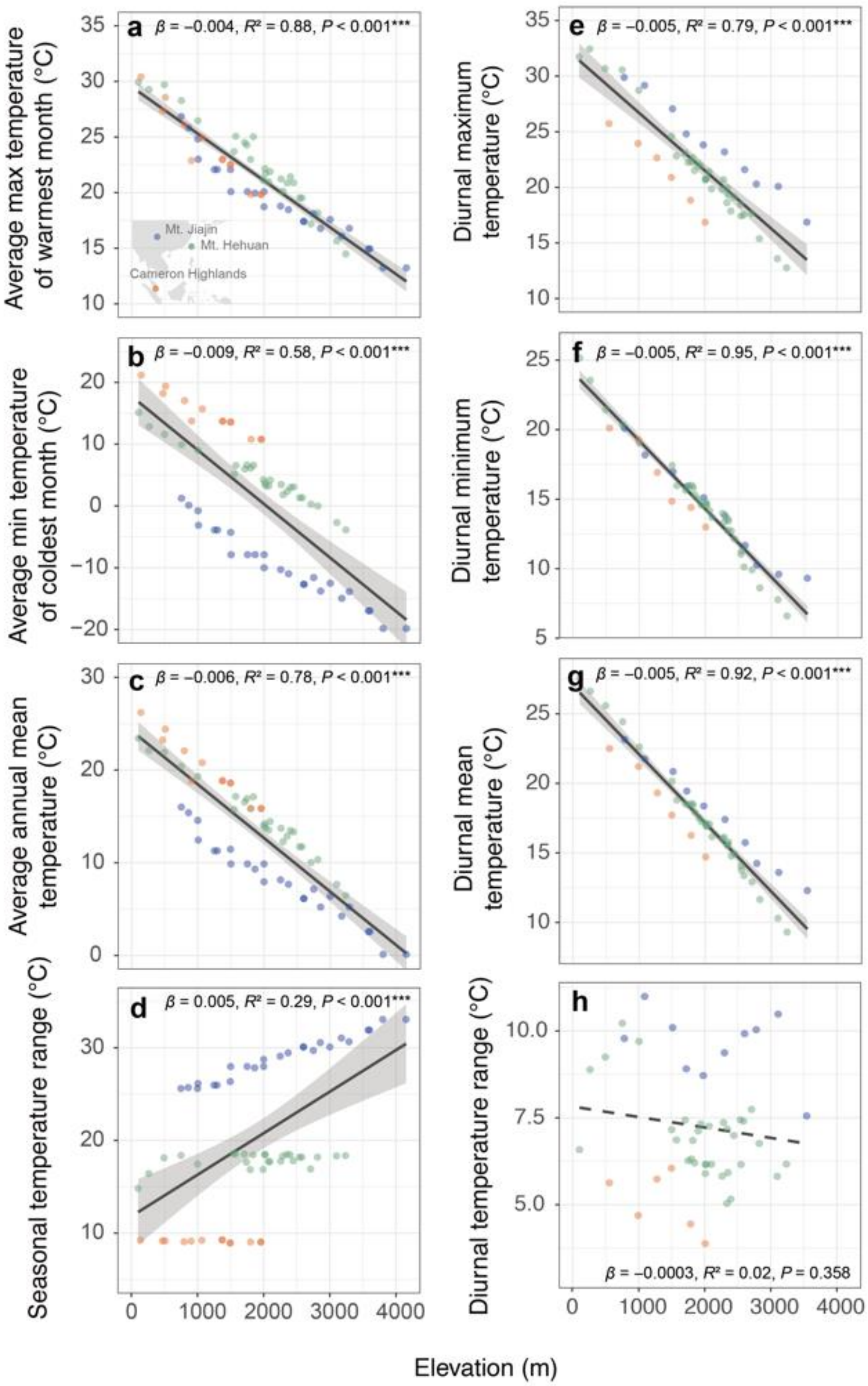

Supplementary Fig. 15. Relationships between ambient seasonal temperature and elevation. Average max temperature of the warmest month (STmax, **a**), average min temperature of the coldest month (STmin, **b**), average annual mean temperature (STmean, **c**), seasonal temperature range (STR, **d**), diurnal maximum temperature (DTmax, **e**), diurnal minimum temperature (DTmin, **f**), diurnal mean temperature (DTmean, **g**), diurnal temperature range (DTR, **h**) in relation to elevation.

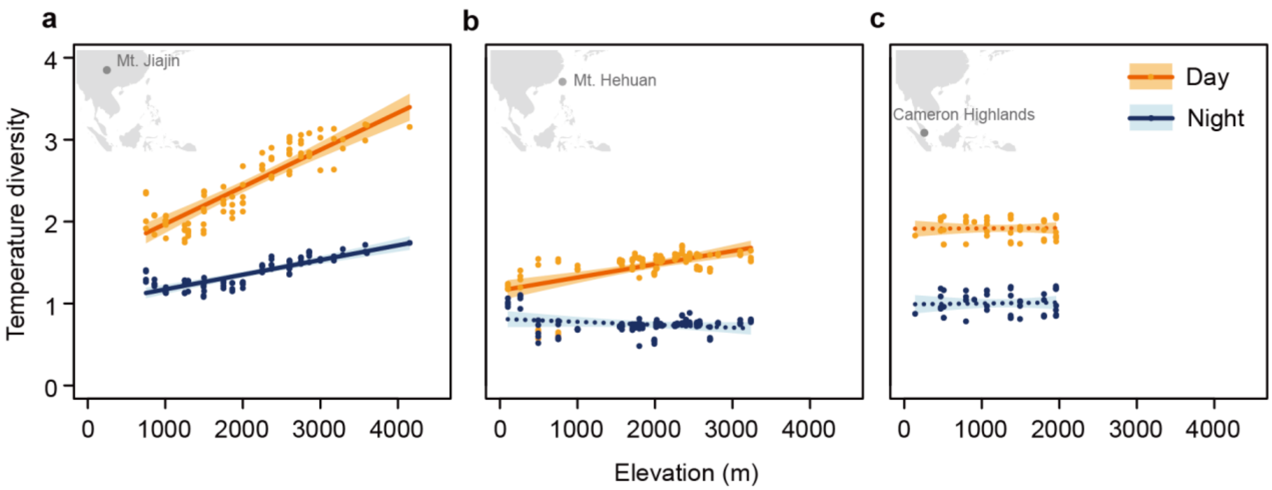

Supplementary Fig. 16. Elevational trends in daytime and nighttime temperature diversity across three geographic regions. Panels show results for Mt. Jiajin (**a**), Mt. Hehuan (**b**), and Cameron Heights (**c**), respectively. Points represent temperature diversity estimates generated using the microclima package. Lines and shaded areas indicate predictions from linear mixed models (LMMs) and their associated 95% confidence intervals. Solid lines denote statistically significant elevational trends ($P < 0.05$), while dotted lines indicate non-significant associations. For full statistical results, see Supplementary Table 10.

Supplementary Tables

Supplementary Table 1. Comparison of predictions among three hypotheses regarding thermal trait diversity at the assemblage level

Aspects	Favorability Hypothesis	Climatic variability hypothesis	Short-term Variability Hypothesis
**Key Environmental Driver**	Mean temperature	Seasonal temperature variation	Daily temperature fluctuation
**Effect on Thermal Trait Diversity**	Higher in warmer environments	Lower in more variable environments	Higher in more variable environments
**Species Composition**	Mix of specialists and generalists	Mainly generalists	Mainly specialists
**Thermal Tolerance Range**	More diverse ranges coexist	Broader ranges dominate	Narrower ranges dominate
**Mechanism**	Higher productivity reduces environmental filtering	Strong selection pressure from temperature variation	Frequent favorable conditions allow specialization
**Primary Prediction**	Thermal trait hypervolume increases with mean temperature	Thermal tolerance range increases with seasonal variation	Thermal specialization increases with daily variation
**Secondary Prediction**	Supports both narrow and broad thermal tolerances	Fewer thermal specialists in variable environments	More thermal specialists in variable environments

Supplementary Table 2. List of parameters and their values used in the eco-evolutionary model.

Parameter name	Value	Description
Thermal parameters		
$T_{env,max}$	38	Critical threshold temperature, above which fecundity is zero for all greater temperature
$T_{env,opt}$	32	The temperature at which the assemblage reaches maximum fecundity
σ_{env}	7.5	Decay coefficient for fecundity, below the optimal temperature
T_{span}	50	Relative time scale difference between short-term and long-term thermal variability
Assemblage parameters		
N_{patch}	200	Number of patches in the assemblage
N_{ini}	3	Number of individuals per species per patch, when initialized
N_{samp}	10	Number of sampling species that produce offspring
$N_{patch\_newspcs}$	10	Number of patches to initialize the immigrated new species
N_{spcs_max}	50	Maximal possible number of species in the assemblage
N_{rep}	50	Number of repetitions for a single environmental setting
Probabilities		
p_{mort}	0.04	Mortality rate for each individual in a time-step
p_{immi}	0.001	Immigration rate in each time-step
Performance curve parameters		
μ_{opt}	25	Average of optimal temperature for generating species' performance curves
σ_{opt}	2.0	Standard deviation of above
μ_{inc}	10	Average of increment from optimal temperature to maximal temperature
σ_{inc}	5	Standard deviation of above
μ_{sgm}	5	Average of decay coefficient for performance below optimal temperature
σ_{sgm}	2.5	Standard deviation of above
Coefficients		
c_{weight}	0.1	Weighting coefficient for density-dependent and density-independent reproduction
c_{fec}	3	Scaling coefficient for fecundity

Supplementary Table 3. Linear models for the analysis of species' thermal trait hypervolumes and the mean and SD of thermal tolerance ranges in relation to ambient temperature gradients in model simulations in Fig. 2. The significance for the linear models and variables was calculated using the two-sided *t*-test with 9 degrees of freedom, respectively. List of abbreviations: Tmean: average annual mean temperature; TTrange: thermal tolerance range.

**Thermal trait hypervolumes**								
**Response variable**	**Predictors**	**Estimate**	**SE**	***t* value**	**CI.low**	**CI.high**	***P* value**	R^2
Thermal traits* hypervolume	Intercept	-0.42	2.00	-0.21	-4.95	4.12	0.84	0.9
	Tmean	1.01	0.11	9.16	0.76	1.26	7.4e-06	
	Intercept	17.94	0.35	50.75	17.1	18.7	2.25e-12	0.04
	Short-term variability	-0.07	0.12	-0.62	-0.34	0.2	0.553	
	Intercept	16.76	0.20	85.50	16.3	17.2	2.08e-14	0.62
	Long-term variability	-0.25	0.07	-3.8	-0.4	-0.1	0.00421	
**Mean and SD of thermal tolerance ranges**								
**Response variable**	**Predictors**	**Estimate**	**SE**	***t* value**	**CI.low**	**CI.high**	***P* value**	R^2
Mean of TTrange	Intercept	37.12	0.68	54.64	35.6	38.7	1.16e-12	0.93
	Tmean	-0.45	0.04	-11.99	-0.53	-0.36	7.74e-07	
SD of TTrange	Intercept	5.84	0.55	10.53	4.59	7.10	2.33e-06	0.54
	Tmean	0.11	0.03	3.46	0.04	0.18	0.00718	
Mean of TTrange	Intercept	29.54	0.19	156.24	29.1	30.0	9.16e-17	0.5
	Short-term variability	-0.20	0.06	-3.15	-0.35	-0.06	0.0117	
SD of TTrange	Intercept	7.82	0.12	67.81	7.56	8.08	1.67e-13	0.04
	Short-term variability	-0.03	0.04	-0.65	-0.11	0.06	0.532	
Mean of TTrange	Intercept	28.32	0.21	134.67	27.8	28.8	3.49e-16	0.89
	Long-term variability	0.65	0.07	9.10	0.49	0.81	7.83e-06	

SD of TTrange	Intercept	7.42	0.06	128.55	7.29	7.55	5.3e-16	0.03
	Long-term variability	0.01	0.02	0.54	-0.03	0.05	0.6	

Note: *Thermal traits include critical thermal maximum and minimum of species.

Supplementary Table 4. Linear models for the analysis of changes of cluster ratio across average annual mean temperature, short-term variability and long-term variability of model simulations in Fig. 3. The significance for the linear models and variables was calculated using the two-sided *t*-test with 9 degrees of freedom, respectively. List of abbreviations: Tmean: average annual mean temperature.

**Average annual mean temperature**								
**Response variable**	**Predictors**	**Estimate**	**SE**	***t* value**	**CI.low**	**CI.high**	***P* value**	**R^2**
Warm-adapted species (red)	Intercept	-0.62	0.03	-23.89	-0.68	-0.57	1.88e-09	0.99
	Tmean	0.05	<0.01	32.93	0.04	0.05	1.08e-10	
Heat-tolerant species (orange)	Intercept	-0.07	0.06	-1.13	-0.2	0.07	0.288025	0.76
	Tmean	0.02	0.003	5.57	0.01	0.03	0.000349	
Cold-adapted species (blue)	Intercept	0.78	0.05	16.64	0.67	0.89	4.56e-08	0.93
	Tmean	-0.03	0.003	-11.65	-0.04	-0.02	9.90e-07	
Heat-sensitive species (green)	Intercept	0.91	0.03	27.03	0.84	0.99	6.29e-10	0.97
	Tmean	-0.04	0.002	-19.12	-0.04	-0.03	1.35e-08	
**Short-term variability**								
**Response variable**	**Predictors**	**Estimate**	**SE**	***t* value**	**CI.low**	**CI.high**	***P* value**	**R^2**
Warm-adapted species (red)	Intercept	0.26	0.01	29.06	0.24	0.28	3.3e-10	0.65
	Short-term variability	0.01	0.003	4.35	0.006	0.02	0.00184	
Heat-tolerant species (orange)	Intercept	0.31	0.01	29.64	0.29	0.33	2.77e-10	0.08
	Short-term variability	-0.003	0.004	-0.92	-0.01	0.005	0.383	
Cold-adapted species (blue)	Intercept	0.21	0.005	44.97	0.197	0.218	6.64e-12	0.57
	Short-term variability	-0.01	0.002	-3.68	-0.009	-0.002	0.00512	
Heat-sensitive species (green)	Intercept	0.22	0.008	27.71	0.201	0.24	5.03e-10	0.21
	Short-term variability	-0.004	0.003	-1.62	-0.01	0.001	0.141	
**Long-term variability**								
**Response variable**	**Predictors**	**Estimate**	**SE**	***t* value**	**CI.low**	**CI.high**	***P* value**	**R^2**

Warm-adapted species (red)	Intercept	0.38	0.01	40.23	0.35	0.4	1.80e-11	0.93
	Long-term variability	-0.04	0.003	-11.72	-0.04	-0.03	9.43e-07	
Heat-tolerant species (orange)	Intercept	0.30	0.007	45.41	0.28	0.31	6.1e-12	0.06
	Long-term variability	0.002	0.002	0.77	-0.003	0.007	0.464	
Cold-adapted species (blue)	Intercept	0.15	0.008	19.12	0.13	0.16	1.35e-08	0.87
	Long-term variability	0.02	0.003	8.21	0.02	0.03	1.80e-05	
Heat-sensitive species (green)	Intercept	0.18	0.006	32.09	0.17	0.19	1.36e-10	0.84
	Long-term variability	0.01	0.002	7.35	0.010	0.018	4.34e-05	

Supplementary Table 5. Linear models analysis for species' thermal trait hypervolumes and linear mixed effects models analysis for the mean and SD of thermal tolerance ranges in relation to ambient temperature gradients in Fig. 4. The significance for the linear models and linear mixed effects models was calculated using the two-sided *t*-test with 7 degrees of freedom and the two-sided Type II Wald chi-square tests, respectively. List of abbreviations: Tmean: average annual mean temperature; DTR: diurnal temperature range; STR: seasonal temperature range.

**Thermal trait hypervolumes**								
**Response variable**	**Predictors**	**Estimate**	**SE**	***t* value**	**CI.low**	**CI.high**	***P* value**	R^2
Thermal traits* hypervolume	Intercept	3.78	2.45	1.54	-2.02	9.57	0.16754	0.67
	Tmean	0.7	0.17	4.07	0.29	1.10	0.00478	
	Intercept	18.7	7.56	2.48	0.83	36.6	0.0425	0.07
	DTR	-0.74	0.94	-0.78	-2.95	1.48	0.4587	
	Intercept	21.44	3.57	6.01	13.0	29.9	0.000535	0.45
	STR	-0.41	0.16	-2.56	-0.78	-0.03	0.0376	

**Mean and SD of thermal tolerance ranges**								
**Response variable**	**Predictors**	**Estimate**	**SE**	**CI.low**	**CI.high**	**Chisq**	***P* value**	R^2
Mean of TTrange	Intercept	42.76	0.75	41.29	44.22	14.87	0.000115	0.65
	Tmean	-0.2	0.05	-0.3	-0.1			
SD of TTrange	Intercept	2.05	0.52	1.04	3.07	6.21	0.01268	0.46
	Tmean	0.09	0.04	0.02	0.16			
Mean of TTrange	Intercept	36.36	1.75	32.93	39.79	4.54	0.03307	0.40
	DTR	0.47	0.22	0.04	0.9			
SD of TTrange	Intercept	-0.22	1.49	-3.15	2.71	8.22	0.004142	0.27
	DTR	0.48	0.17	0.15	0.8			
Mean of TTrange	Intercept	36.80	0.57	35.68	37.93	38.1	6.737e-10	0.83
	STR	0.16	0.03	0.11	0.21			
SD of TTrange	Intercept	4.08	0.62	2.86	5.29	2.25	0.1337	0.22
	STR	-0.04	0.03	-0.096	0.013			

Note: *Thermal traits include critical thermal maximum and minimum of species.

Supplementary Table 6. Linear mixed effects models for the analysis of changes of cluster ratio across average annual mean temperature (a), diurnal temperature range (b) and seasonal temperature range (c) of empirical data in Fig. 5. The significance for the linear models and variables was calculated using the two-sided Type II Wald chi-square tests. List of abbreviations: Tmean: average annual mean temperature; DTR: diurnal temperature range; STR: seasonal temperature range.

**Average annual mean temperature**							
**Response variable**	**Predictors**	**Estimate**	**SE**	**Chisq**	**Df**	***P* value**	**R^2**
Warm-adapted species (red)	Intercept	-0.21	0.10	16.84	1	4.057e-05	0.63
	Tmean	0.02	0.006				
Heat-tolerant species (orange)	Intercept	-0.57	0.24	55.05	1	1.175e-13	0.52
	Tmean	0.06	0.009				
Cold-adapted species (blue)	Intercept	1.23	0.22	25.97	1	3.466e-07	0.69
	Tmean	-0.06	0.01				
Heat-sensitive species (green)	Intercept	0.37	0.19	1.14	1	0.2867	0.11
	Tmean	-0.01	0.01				
**Diurnal temperature range**							
**Response variable**	**Predictors**	**Estimate**	**SE**	**Chisq**	**Df**	***P* value**	**R^2**
Warm-adapted species (red)	Intercept	0.15	0.18	0.05	1	0.8277	0.01
	DTR	-0.005	0.02				
Heat-tolerant species (orange)	Intercept	0.13	0.34	0.37	1	0.5418	0.04
	DTR	0.03	0.04				
Cold-adapted species (blue)	Intercept	0.16	0.44	0.23	1	0.6292	0.03
	DTR	0.03	0.05				
Heat-sensitive species (green)	Intercept	0.58	0.21	3.58	1	0.05858	0.35
	DTR	-0.05	0.03				
**Seasonal temperature range**							
**Response variable**	**Predictors**	**Estimate**	**SE**	**Chisq**	**Df**	***P* value**	**R^2**
Warm-adapted species (red)	Intercept	0.25	0.10	2.28	1	0.1311	0.22
	STR	-0.01	0.004				

Heat-tolerant species (orange)	Intercept	0.44	0.22	0.27	1	0.6023	0.03
	STR	-0.005	0.010				
Cold-adapted species (blue)	Intercept	-0.05	0.22	3.9	1	0.04828	0.33
	STR	0.02	0.01				
Heat-sensitive species (green)	Intercept	0.36	0.13	1.79	1	0.1815	0.19
	STR	-0.01	0.01				

Supplementary Table 7. Type III ANOVA table for the full model of linear mixed model (LMM) results evaluating regional variation in the elevational trends of temperature diversity during daytime and nighttime. The significance for the linear models and variables was calculated using the two-sided Type III Wald chi-square tests.

	**Chi-square**	**df**	**P**
Intercept	1301	1	<0.001
Elevation	644	1	<0.001
Region	147	2	<0.001
Period	137	1	<0.001
Elevation*Region	207	2	<0.001
Elevation*Period	250	1	<0.001
Region*Period	79	2	<0.001
Elevation*Region*Period	53	2	<0.001

N=526; marginal R^2=0.95; conditional R^2=0.97

Supplementary Table 8. Pairwise post hoc comparisons among regions in temperature diversity during daytime and nighttime. The comparisons are made after the linear mixed model in Supplementary Table 7. P-values (two-sided) are obtained with Tukey method for multiple comparison adjustment and Kenward-Roger approximation for degrees of freedom. P-values smaller than 0.0001 are reported as <0.0001 (default setting of the *emmeans* package in R).

Period	Contrast	Estimate	SE	df	T ratio	P
Day	Sichuan - Malaysia	0.44	0.03	412	12.8	<0.0001
	Sichuan - Taiwan	0.90	0.02	412	44.6	<0.0001
	Malaysia - Taiwan	0.47	0.03	412	14.1	<0.0001
Night	Sichuan - Malaysia	0.32	0.03	412	9.2	<0.0001
	Sichuan - Taiwan	0.59	0.03	412	28.9	<0.0001
	Malaysia - Taiwan	0.27	0.03	412	8.1	<0.0001

Supplementary Table 9. Pairwise post hoc comparisons between daytime and nighttime in temperature diversity within regions. The comparisons are made after the linear mixed model in Supplementary Table 7. P-values (two-sided) are obtained with Tukey method for multiple comparison adjustment and Kenward-Roger approximation for degrees of freedom. P-values smaller than 0.0001 are reported as <0.0001 (default setting of the *emmeans* package in R).

Region	Contrast	Estimate	SE	df	T ratio	P
Sichuan	Day - Night	1.03	0.02	257	65.5	<0.0001
Taiwan	Day - Night	0.71	0.01	257	55.6	<0.0001
Malaysia	Day - Night	0.91	0.03	257	29.8	<0.0001

Supplementary Table 10. Elevational trends in temperature diversity within each region and diel period estimated using the *emtrends* function. The trends are estimated from the linear mixed model in Supplementary Table 7. P-values (two-sided) are obtained with Kenward-Roger approximation for degrees of freedom. P-values smaller than 0.001 are reported as <0.001 (default setting of the *emmeans* package in R).

Region	Period	Elevation	SE	df	T ratio	P
Mt. Jiajin	Day	4.75e-04	1.87e-05	412	25.4	<0.0001
	Night	1.78e-04	1.87e-05	412	9.5	<0.0001
Mt. Hehuan	Day	1.65e-04	1.66e-05	412	9.9	<0.0001
	Night	-2.92e-05	1.66e-05	412	-1.8	0.08
Cameron Heights	Day	3.97e-06	3.88e-05	412	0.1	0.92
	Night	1.13e-05	3.88e-05	412	0.3	0.77

Supplementary References

1 Via, S. & Lande, R. Genotype-Environment Interaction and the Evolution of Phenotypic Plasticity. *Evolution* **39**, 505-522 (1985). https://doi.org/10.2307/2408649

2 Scheiner, S. M. The genetics of phenotypic plasticity. XII. Temporal and spatial heterogeneity. *Ecology and Evolution* **3**, 4596-4609 (2013). https://doi.org/10.1002/ece3.792
