# Supplementary material for: Warmer environments harbor greater thermal trait diversity in moth assemblages
Source: Nat Commun. 2025 Dec 11;17:738. doi: 10.1038/s41467-025-67419-8 (PMC12820197; doi:10.1038/s41467-025-67419-8)
Supplement: Supplementary file 2 — Reporting Summary [file 41467_2025_67419_MOESM2_ESM.pdf]

nature portfolio

Corresponding author(s): Sheng-Feng Shen, I-Ching Chen

Last updated by author(s): Nov 21, 2025

Reporting Summary

Nature Portfolio wishes to improve the reproducibility of the work that we publish. This form provides structure for consistency and transparency in reporting. For further information on Nature Portfolio policies, see our Editorial Policies and the Editorial Policy Checklist.

Please do not complete any field with "not applicable" or n/a. Refer to the help text for what text to use if an item is not relevant to your study.
For final submission: please carefully check your responses for accuracy; you will not be able to make changes later.

Statistics

For all statistical analyses, confirm that the following items are present in the figure legend, table legend, main text, or Methods section.

n/a	Confirmed	
☐	☒	The exact sample size (*n*) for each experimental group/condition, given as a discrete number and unit of measurement
☒	☐	A statement on whether measurements were taken from distinct samples or whether the same sample was measured repeatedly
☐	☒	The statistical test(s) used AND whether they are one- or two-sided<br>*Only common tests should be described solely by name; describe more complex techniques in the Methods section.*
☒	☐	A description of all covariates tested
☒	☐	A description of any assumptions or corrections, such as tests of normality and adjustment for multiple comparisons
☐	☒	A full description of the statistical parameters including central tendency (e.g. means) or other basic estimates (e.g. regression coefficient) AND variation (e.g. standard deviation) or associated estimates of uncertainty (e.g. confidence intervals)
☐	☒	For null hypothesis testing, the test statistic (e.g. *F*, *t*, *r*) with confidence intervals, effect sizes, degrees of freedom and *P* value noted<br>*Give P values as exact values whenever suitable.*
☒	☐	For Bayesian analysis, information on the choice of priors and Markov chain Monte Carlo settings
☒	☐	For hierarchical and complex designs, identification of the appropriate level for tests and full reporting of outcomes
☒	☐	Estimates of effect sizes (e.g. Cohen's *d*, Pearson's *r*), indicating how they were calculated

Our web collection on statistics for biologists contains articles on many of the points above.

Software and code

Policy information about availability of computer code

Data collection	No custom algorithms or modeling software were used during field data acquisition. Thermal images were captured with a FLIR T420 and processed with ThermaCAM Researcher Pro v2.10 (commercial software, FLIR Systems) to extract thoracic temperatures; the camera was factory-calibrated. iButton loggers (Maxim Integrated) were configured and downloaded with the manufacturer’s tools (OneWire/iButton Viewer) to record temperatures at 30-min intervals and export CSV files. Apart from these instrument utilities, no other commercial, open-source, or custom software was used at the data-collection stage.
Data analysis	Analyses used open-source software and custom code. A custom C program implemented the eco-evolutionary simulation framework that generated derived data (e.g., critical thermal limits). R 4.3.0 was used with the following packages: "hypervolume" (v3.1.6), "ks" (for kernel density estimation; v1.14.3), base "kmeans" (thermal-strategy clustering; v4.3.0), "lme4" (linear mixed-effects models; v1.1.36), "emmeans" (Tukey tests and slope comparisons; v1.8.8), "mcera5" (ERA5 hourly data retrieval; v0.4.0), and "microclima" (microclimate modeling; v0.1.0). Python 3.11.5 was used with "scikit-learn" (random-forest regression; v1.3.0) and "shap" (SHAP value computation and interpretation; v0.42.1). All the analysis code used in this study has been deposited in the Zenodo repository under the accession code 17409650 (https://doi.org/10.5281/zenodo.17409650).

For manuscripts utilizing custom algorithms or software that are central to the research but not yet described in published literature, software must be made available to editors and reviewers. We strongly encourage code deposition in a community repository (e.g. GitHub). See the Nature Portfolio guidelines for submitting code & software for further information.

Data

Policy information about availability of data

All manuscripts must include a data availability statement. This statement should provide the following information, where applicable:

- Accession codes, unique identifiers, or web links for publicly available datasets
- A description of any restrictions on data availability
- For clinical datasets or third party data, please ensure that the statement adheres to our policy

All data generated or used in this study have been deposited in the Zenodo repository under the accession code 17409650 (https://doi.org/10.5281/zenodo.17409650).

Research involving human participants, their data, or biological material

Policy information about studies with human participants or human data. See also policy information about sex, gender (identity/presentation), and sexual orientation and race, ethnicity and racism.

Reporting on sex and gender	This study did not collect any human participant data related to sex or gender.
Reporting on race, ethnicity, or other socially relevant groupings	This study did not collect any human race, ethnicity, or other socially relevant groupings information.
Population characteristics	No human populations characteristics were involved in this research.
Recruitment	This study did not involve any human participant recruitment.
Ethics oversight	This study did not require human subjects ethics review.

Note that full information on the approval of the study protocol must also be provided in the manuscript.

Field-specific reporting

Please select the one below that is the best fit for your research. If you are not sure, read the appropriate sections before making your selection.

☐ Life sciences ☐ Behavioural & social sciences ☒ Ecological, evolutionary & environmental sciences

For a reference copy of the document with all sections, see nature.com/documents/nr-reporting-summary-flat.pdf

Ecological, evolutionary & environmental sciences study design

All studies must disclose on these points even when the disclosure is negative.

Study description	This study combined theoretical modeling with field measurements to investigate thermal trait diversity across elevational gradients. We developed an eco-evolutionary model to test three hypotheses (favorability,long-term variability, and short-term variability) and conducted field experiments measuring critical thermal limits of moths across three elevational gradients in Asia. The experimental design included sampling stations established at approximately 500m elevation intervals, ranging from 140m to 4,150m above sea level.
Research sample	The study examined 653 moth species collected across three Asian elevation gradients: Cameron Heights, Malaysia (140-1,959m), Mt. Hehuan, Taiwan (343-3,140m), and Mt. Jiajin, Sichuan, China (860-4,150m). In total, we assessed 1,475 individuals, 16 families in Malaysia, 2,257 individuals, 14 families in Taiwan, and 1,917 individuals, 15 families in China.
Sampling strategy	Thermal testing stations were established at 500m elevation intervals along each transect. Sample sizes were not predetermined statistically but were based on comprehensive sampling of moth species present at each elevation. Moths were attracted using a 100-watt lamp and white screen at each station. The large sample sizes (>1,400 individuals per location) provided sufficient statistical power to detect thermal trait patterns across environmental gradients.
Data collection	Critical thermal limits (CTmax and CTmin) were measured using hot-water (50°C) and cold-water (-5°C) baths, with thermal responses monitored via FLIR T420 camera. Temperature data were recorded when moths exhibited loss of muscle control or spasms. Thorax temperatures were analyzed using ThermaCAM researcher 2.10, with values rounded to the first decimal place. Data collected was conducted mainly by Shiper Wu, who both led field experiments and did taxonomic identification.
Timing and spatial scale	Data collection was conducted during: July 24–August 21, 2019 in Cameron Heights, Malaysia, elevation spanning from 140 to 1,959m; July 20–August 28, 2015 in Mt. Hehuan, Taiwan, spanning from 343 to 3,140m; and June 28–July 22, 2017 in Mt. Jiajin, Sichuan, spanning from 860 to 4,150m.
Data exclusions	No data were excluded from the analyses. All species with both CTmax and CTmin measurements were included in the study.

Reproducibility	The theoretical model simulations were repeated 50 times for each environmental setting to ensure robust results. Every hypervolume result is average by 100 repeats as well.
Randomization	This study examined natural moth populations across elevational gradients and did not involve experimental group allocation. Environmental variables were natural gradients rather than manipulated treatments.
Blinding	Blinding was not relevant to this study as it involved objective measurements of physiological responses (critical thermal limits) using standardized protocols and thermal imaging equipment.

Did the study involve field work? ☒ Yes ☐ No

Field work, collection and transport

Field conditions	Field work was conducted during summer months across three locations. Temperature conditions varied across elevation gradients: Cameron Heights (July 24-August 21, 2019, mean temperature spans from 25°C at low elevation to 15°C at high elevation), Mt. Hehuan (July 20-August 28, 2015, mean temperature spans from 8 to 21°C), and Mt. Jiajin (June 28-July 22, 2017, mean temperature spans from 1 to 16°C). Temperature was monitored continuously using iButton thermometers recording at 30-minute intervals.
Location	The study was conducted at three locations in Asia: (1) Cameron Heights, Malaysia (4°28'0''–4°36'0''N, 101°11'0''–101°23'0''E; elevation: 140-1,959m.) (2) Mt. Hehuan, Taiwan (24°16'0''–24°21'0''N, 121°10'0''–121°40'0''E; elevation: 343-3,140m.) (3) Mt. Jiajin, Sichuan, China (30°23'0''–30°85'0''N, 102°69'0''–102°90'0''E; elevation: 860-4,150m.)
Access & import/export	For Cameron Heights, Malaysia, we obtained the necessary collection permit issued by Economic Planning Unit, Prime Minister's Department of Malaysia (reference number: UPE 40/200/19/3529), valid from April 25, 2019 to April 24, 2020. For Mt. Hehuan, Taiwan, sampling was conducted under permit No. 1040012350 issued by Taroko National Park, valid from June 10, 2015 to December 31, 2015. For Mt. Jiajin, Sichuan, China, no specific permit was required as the sampled species were not protected under local regulations.
Disturbance	Field sampling was conducted using light trapping methods that minimized habitat disturbance. Sampling stations were established along existing trails to avoid artificial buildings or facilities. Moths were placed individually for calming down and the further experiment after captured, and carefully handled during thermal tolerance testing to minimize stress. After testing, specimens were preserved following standard museum protocols.

Reporting for specific materials, systems and methods

We require information from authors about some types of materials, experimental systems and methods used in many studies. Here, indicate whether each material, system or method listed is relevant to your study. If you are not sure if a list item applies to your research, read the appropriate section before selecting a response.

Materials & experimental systems

n/a	Involved in the study
☒	☐ Antibodies
☒	☐ Eukaryotic cell lines
☒	☐ Palaeontology and archaeology
☐	☒ Animals and other organisms
☒	☐ Clinical data
☒	☐ Dual use research of concern
☒	☐ Plants

Methods

n/a	Involved in the study
☒	☐ ChIP-seq
☒	☐ Flow cytometry
☒	☐ MRI-based neuroimaging

Animals and other research organisms

Policy information about studies involving animals; ARRIVE guidelines recommended for reporting animal research, and Sex and Gender in Research

Laboratory animals	This study did not involve laboratory animals. All specimens were collected from natural field environments.
Wild animals	Moths used in this study were attracted using a 100-watt lamp and white screen at each sampling station. Upon capture, specimens were subjected to thermal tolerance testing and were subsequently preserved in freezer for further taxonomic identification. All specimens were deposited at the Biodiversity Research Museum, Academia Sinica, Taipei, Taiwan.
Reporting on sex	Sex was not considered as a variable in this study of moth thermal tolerances. The study focused on species-level thermal trait patterns across environmental gradients, and sex-based differences were not within the scope of this research.
Field-collected samples	This study did not contain laboratory works with field-collected samples.

Ethics oversight	No specific ethical approval was required for this invertebrate study.

Note that full information on the approval of the study protocol must also be provided in the manuscript.

Plants

Seed stocks	This study did not contain plant samples.
Novel plant genotypes	This study did not contain plant samples.
Authentication	This study did not contain plant samples.

This checklist template is licensed under a Creative Commons Attribution 4.0 International License, which permits use, sharing, adaptation, distribution and reproduction in any medium or format, as long as you give appropriate credit to the original author(s) and the source, provide a link to the Creative Commons license, and indicate if changes were made. The images or other third party material in this article are included in the article's Creative Commons license, unless indicated otherwise in a credit line to the material. If material is not included in the article's Creative Commons license and your intended use is not permitted by statutory regulation or exceeds the permitted use, you will need to obtain permission directly from the copyright holder. To view a copy of this license, visit http://creativecommons.org/licenses/by/4.0/
